# Supplementary material for: Intraspecific body size variation and allometry of genitalia in the orb-web spider—Argiope lobata
Source: PeerJ. 2023 Nov 28;11:e16413. doi: 10.7717/peerj.16413 (PMC10691382; doi:10.7717/peerj.16413)
Supplement: Table SA1 [file peerj-11-16413-s002.docx]

**Appendix 1**

**Table A1:** Summary statistics of spider traits used in the analysis. Linear units are µm and area units are µm^2^.

| Trait | Mean | SD | CV | n |
| --- | --- | --- | --- | --- |
| *Female TPL* | 10319.7 | 660.6 | 0.064 | 50 |
| $\sqrt{\boldsymbol{Female carapace area}}$ | 6338.9 | 358.1 | 0.056 | 50 |
| *Median septum length* | 713.3 | 56.7 | 0.08 | 50 |
| *Median septum width* | 825.0 | 82.8 | 0.10 | 50 |
| *Male TPL* | 3923.2 | 400.9 | 0.10 | 45 |
| $\sqrt{\boldsymbol{Male carapace area}}$ | 2456.1 | 177.2 | 0.072 | 47 |
| *Pedipalp apophysis area* | 94529.9 | 8725.2 | 0.092 | 46 |
| *Spur length* | 341.9 | 32.5 | 0.095 | 46 |
