# Supplementary material for: Intraspecific body size variation and allometry of genitalia in the orb-web spider—Argiope lobata
Source: PeerJ. 2023 Nov 28;11:e16413. doi: 10.7717/peerj.16413 (PMC10691382; doi:10.7717/peerj.16413)
Supplement: Supplemental Information 3 [file peerj-11-16413-s003.docx]

## **Appendix 1**

## **Alternative analysis of allometric relationships**

When analysing allometric relationships, we chose to use tibia-patella length as our indicator of spider body size. However, the square root of carapace area may be a better linear indicator of body size for sexually dimorphic species such as *Argiope lobata*. Accordingly, we here perform the same allometric relationship tests as in the main article, using square root of carapace area rather than tibia-patella length as our indicator body size. We estimated carapace area as carapace width × height.

Of the four traits we compared, only a single RMA slope differed significantly from 1: female median septum width, with a slope >1 (Table S1, Figure S1). Of the four traits, only pedipalp apophysis area and $\sqrt{carapace area}$showed a significant correlation. We required both a significant positive correlation and an RMA slope <> 1 to establish a non-isometric relationship, and none of the relationship we tested satisfied both criteria. These results are qualitatively the same as our results when using tibia-patella length as the indicator of body size. Accordingly, our conclusions are not affected by our choice of body size indicator.


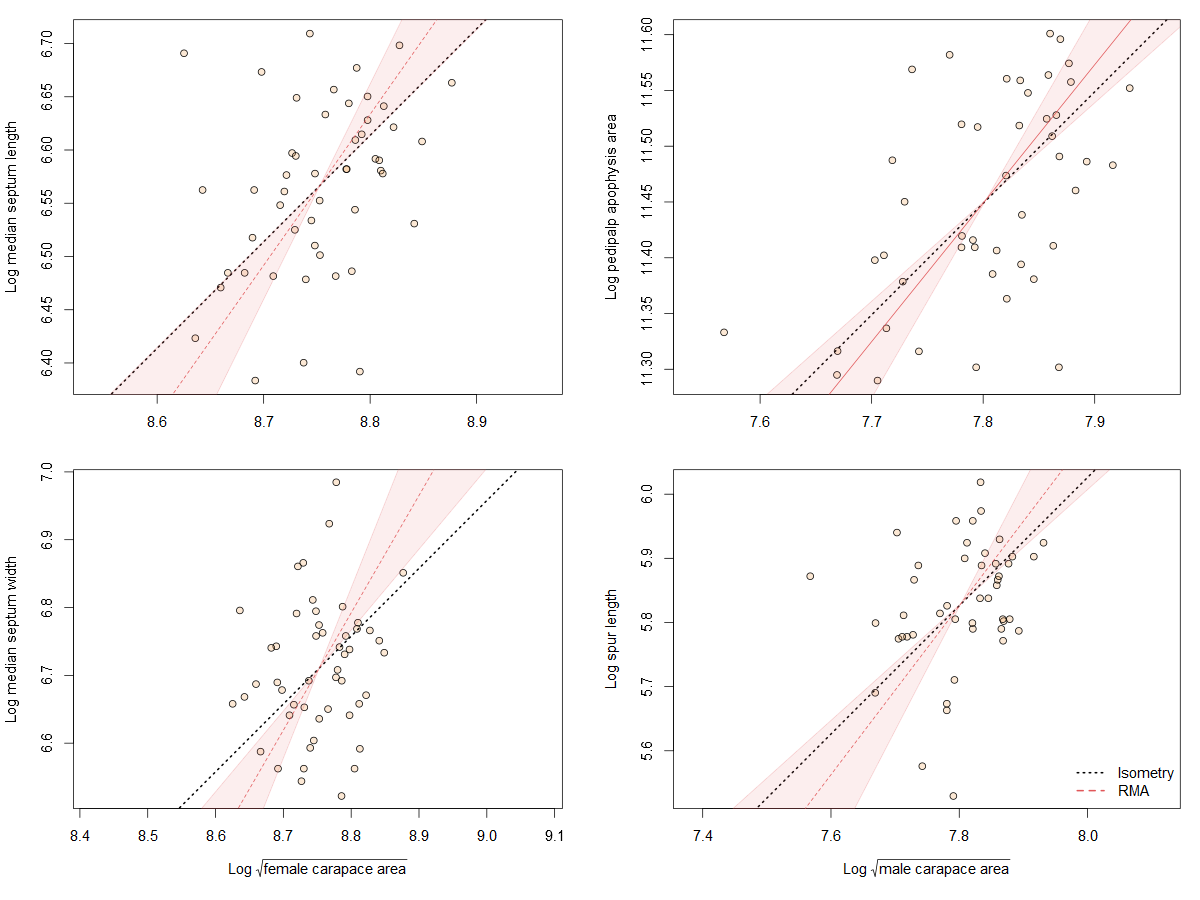


Figure S1.Static allometry of genital structures and body size proxy ($\sqrt{carapace area}$) in female (left panels) and male (right panels) A. lobata. Red line shows the fitted RMA regression; dashed indicates there is not a significant correlation between the two variables. Filled polygon depicts 99% confidence intervals (CI) of the RMA slope. The black dotted line indicates isometry (slope = 1 and passing through the centroid of the points). Points represent individual spiders. Evidence for allometry requires 1) a significant correlation between the two variables and 2) the RMA CI does not include the line of isometry. None of the relationships satisfy both conditions.

Table S1. Static allometry: 99% confidence interval (lower limit, LL, and upper limit, UL) of slope of reduced major axis (RMA) regression analysis and correlation (P-value and R^2^) of male and female genital characteristics on body size proxy ($\sqrt{carapace area}$, SCA) in A. lobata. LL and UL values in bold indicate slope ≠ 1. P-value and R^2^ in bold indicate a significant positive correlation between SCA and genital trait at the p<0.01 level

| Body size vs genital characteristics | LL | UL | P | R^2^ | n |
| --- | --- | --- | --- | --- | --- |
| *Median septum length vs female SCA* | 0.99 | 2.02 | 0.013 | 0.12 | 50 |
| *Median septum width vs female SCA* | **1.19** | **2.51** | 0.023 | 0.03 | 50 |
| *Pedipalp apophysis area vs male SCA* | 0.89 | 1.73 | **<0.001** | **0.29** | 47 |
| *Spur length vs male SCA* | 0.89 | 1.93 | 0.066 | 0.07 | 47 |
